# Fragmented prevention in rural South Africa: a qualitative study of Biokineticists’ perspectives on health system barriers to early detection of non-communicable diseases

**DOI:** 10.1080/16549716.2026.2707659

**Published:** 2026-07-31

**Authors:** Gudani G. Mukoma, Lisa K. Micklesfield, Estelle D. Watson, Shane A. Norris

**Affiliations:** a Department of Biokinetics, Recreation and Sport Science, Faculty of Health Science, University of Venda, Thohoyandou, South Africa; b SAMRC/ Wits Ageing African Adult Research Unit, Department of Paediatrics, Faculty of Health Sciences, School of Clinical Medicine, University of the Witwatersrand, Johannesburg, South Africa; c School of Exercise, Sport and Rehabilitation Sciences, Faculty of Science, University of Auckland, Auckland, New Zealand; d School of Human Development and Health, University of Southampton, Southampton, UK

**Keywords:** Biokinetics, allied health, early detection, rural health, South Africa

## Abstract

**Background:**

Non-communicable diseases (NCDs) disproportionately affect rural South Africans due to limited healthcare access and delayed diagnoses. Biokineticists, experts in clinical exercise therapy, can aid in the early detection and prevention of NCDs, but their integration into primary healthcare remains limited.

**Objective:**

To explore Biokineticists’ perspectives on their role in early NCD detection and prevention in rural South Africa, along with health-system factors affecting their integration into public primary healthcare.

**Methods:**

An interpretivist qualitative study using semi-structured interviews was conducted with 10 purposively selected Health Professions Council of South Africa (HPCSA)-registered Biokineticists (6 female, 4 male; median age 34; median 10 years’ practice experience). Interviews explored screening, health promotion, referrals, interprofessional collaboration, and systemic barriers. Sessions were recorded, transcribed, and analysed using Braun and Clarke‘s thematic analysis.

**Results:**

Four interconnected domains were identified: (1) professional identity and recognition, (2) screening and early-detection practices, (3) community-based prevention and management, and (4) structural barriers and integration opportunities. Participants conducted screenings, exercise-based interventions, and health promotion activities, but faced fragmented referral pathways, low awareness of Biokinetics, limited visibility, and financial and geographic barriers affecting rural access. While participants viewed Biokineticists as capable contributors to NCD prevention and early detection, systemic issues, including weak integration into primary healthcare and the National Health Insurance framework (NHI), hindered continuity of care. Conclusion: Biokineticists are an underused allied-health resource with the potential to strengthen early detection and prevention of NCDs in rural primary care settings; addressing structural and policy barriers is crucial for their integration into primary care.

## Paper Context

- **Main findings:** Biokineticists demonstrate capacity for early detection of NCD risk factors, but their contribution is constrained by fragmented health system structures that limit continuity of care.
- **Added knowledge:** This study provides qualitative evidence on how structural, professional, and financing barriers shape the limited integration of Biokinetics within primary healthcare in rural South Africa.
- **Global health impact for policy and action:** Addressing structural and financing barriers to integrating prevention-oriented allied health professionals within primary healthcare systems may enhance early detection and continuity of care for NCDs in low- and middle-income settings.

## Background

Non-communicable diseases (NCDs), such as cardiovascular diseases, diabetes, chronic respiratory diseases, and cancers, account for about 75% of the global deaths, with the greatest burden falling on low- and middle-income countries (LMICs) [1,2]. In sub-Saharan Africa, this burden overlaps with infectious diseases, placing additional strain on already resource-constrained health systems [3,4]. Rural populations are disproportionately affected because they experience shortages of healthcare professionals, inadequate diagnostic infrastructure, long travel distances, financial barriers, and fragmented referral pathways that delay diagnosis and compromise continuity of care [5–7]. Rural populations are disproportionately affected because of shortages of healthcare professionals, limited diagnostic infrastructure, long travel distances, financial barriers, and fragmented referral pathways that delay diagnosis and compromise continuity of care [1]. As a result, early detection has become a key global public health strategy, although equitable access remains difficult in rural and underserved communities.

Addressing this challenge requires stronger primary healthcare (PHC) systems supported by multidisciplinary care rather than physician-led services alone. Allied health professionals contribute to NCD prevention by promoting healthy lifestyles, identifying at-risk individuals, supporting behaviour change, and providing rehabilitation [8–10]. International organisations, including the World Health Organization (WHO), recommend integrating appropriately trained allied health professionals into PHC to improve equitable access to preventive services and strengthen health-system resilience, particularly in underserved settings [11,12].

Among these allied health professions, exercise professionals are increasingly recognised for their contribution to chronic disease prevention and management [8]. Although their professional titles vary worldwide, their clinical roles are similar. They include Clinical Exercise Physiologists in Australia and the United States, Registered Kinesiologists in Canada, and Clinical Exercise Scientists or Exercise Rehabilitation Specialists in Europe. They prescribe evidence-based exercises to prevent and manage cardiovascular disease, diabetes, obesity, musculoskeletal and neurological conditions, improving physical function, reducing risk, and enhancing quality of life [13,14]. Growing evidence also shows that integrating exercise professionals into multidisciplinary teams improves outcomes, continuity of care, and long-term cost savings [14].

In South Africa, Biokinetics is a regulated allied health profession registered with the HPCSA. Biokineticists complete at least 4 years of professional training and specialise in the scientific use of exercise for health promotion, disease prevention, rehabilitation, and improving functional capacity in individuals with chronic diseases and disabilities [15–17]. Despite evidence supporting exercise therapy in hypertension, diabetes, and obesity, Biokinetic services remain concentrated in the private sector, with limited representation in the public PHC system [15,17]. This leaves rural populations, who bear a high burden of NCDs, with limited access to services that could support prevention, early detection, and long-term management.

South Africa‘s National Strategic Plan for the Prevention and Control of NCDs (2022–2027) prioritises prevention, early detection, and integrated chronic disease management [18]. The implementation of the NHI similarly aims to improve equitable access to comprehensive PHC services [19,20]. These priorities align with WHO recommendations for multidisciplinary care and greater inclusion of allied health professionals in PHC [9,11,18]. However, Biokineticists remain largely excluded from routine public-sector PHC services, limiting their contribution to population-level prevention and early detection of NCDs in rural South Africa.

This study was informed by Levesque et al.‘s conceptual framework of access to healthcare [21], which views access as the interaction between health-system characteristics and people‘s abilities to obtain appropriate care. The framework is useful for understanding how organisational, professional, and health-system factors influence the integration of Biokinetics into rural PHC and the delivery of preventive services.

Although previous studies highlight Biokineticists’ role in chronic disease management and workforce challenges in South Africa [15–17], little research has explored their perspectives on rural NCD prevention, early detection, or the health-system factors shaping their integration into PHC. Existing work has focused mainly on workforce distribution, professional recognition, and clinical effectiveness, with limited attention to the experiences of delivering preventive services in underserved rural communities [15–17,22]. Understanding these perspectives is essential for informing workforce planning, strengthening multidisciplinary PHC, and identifying practical strategies for integrating exercise-based allied health services into rural healthcare systems.

This study explored Biokineticists’ views on their role in the early detection and prevention of NCDs in rural South Africa, alongside the health-system factors affecting their integration into public PHC. It asked what Biokineticists perceive their role to be in early NCD detection and prevention, and which health-system factors influence their integration? The objectives were to explore perceptions of their professional role, describe current screening, referral, and health-promotion practices, identify organisational, socio-cultural, and system barriers in rural service delivery, and identify opportunities to strengthen integration into multidisciplinary PHC.

## Materials and methods

### Study design and theoretical orientation

This study employed a qualitative design, using in-depth interviews (IDIs), to explore Biokineticists’ perspectives on their current and potential roles in rural NCD detection and prevention, and as well as their experiences of system-level constraints affecting service delivery in rural South Africa. The research was underpinned by an interpretivist paradigm [23], recognising that professional roles and health system integration are socially constructed and shaped by contextual experiences. IDIs were selected to facilitate detailed exploration of individual perspectives, professional identity, and experiences of system-level barriers that may not emerge in group-based discussions. This study is reported in accordance with the Consolidated Criteria for Reporting Qualitative Research (COREQ), and the completed checklist is provided as supplementary file 1.

### Study setting and context

The study was conducted within the broader context of South Africa‘s two-tiered healthcare system, characterised by a well-resourced private sector and a public sector serving the majority of the population [24]. Rural and underserved areas face persistent structural challenges, including shortages of healthcare professionals, limited diagnostic infrastructure, geographic barriers, and financial constraints [25]. Biokineticists predominantly practise in private-sector or military healthcare settings, with minimal representation in public PHC settings. Rural South Africa was selected because these communities experience a disproportionate burden of NCDs, persistent shortages of allied health professionals and limited access to preventive rehabilitation services, making the setting particularly relevant for exploring the integration of Biokinetics into PHC.

### Participant recruitment and sampling

A purposive sampling strategy was employed to recruit Biokineticists with experience in rural outreach and/or public-sector healthcare contexts across provinces in South Africa. Inclusion criteria required participants to:

- Be registered with the Health Professions Council of South Africa (HPCSA) as a Biokineticist
- Have experience delivering services in rural, outreach, or public-sector settings
- Have at least 2 years of professional practice experience

Participants were excluded if they:

- Were not registered with the HPCSA;
- Had fewer than 2 years’ professional experience;
- Had no experience in rural outreach or public-sector practice;
- Inability to provide written informed consent;
- Declined audio recording

Participants were identified through professional networks, university alumni databases, and snowball referrals. Sampling aimed for maximum variation across gender, geographic location, and sector (public/private) while purposively recruiting information-rich participants with substantial experience in rural outreach and/or public-sector practice. This approach was considered essential for generating in-depth insights into organisational and health-system factors influencing the integration of Biokinetics within rural PHC. Recruitment continued until thematic saturation was reached, defined as the point at which successive interviews yielded no substantively new codes or themes. Saturation was assessed iteratively during data collection through concurrent preliminary coding and discussion among the research team. A total of 10 Biokineticists (6 female and 4 male) participated in this study.

### Data collection

Data were collected by the first author (GGM), a male Biokineticist, lecturer, and NRF-rated researcher with a doctorate in Health Sciences and experience in qualitative interviews within community and health systems research. Participants were informed of the interviewer‘s background and the study aim before the interview. Although no personal relationships existed, some participants were known professionally through the Biokinetics community. The IDIs took place from May to June 2025, either in person or via a secure online platform (Microsoft Teams), based on location and logistics. The semi-structured interview guide (Appendix 1) was developed from the study objectives and relevant literature and was reviewed by two qualitative researchers and one senior Biokineticist with expertise in NCD prevention to establish content relevance and clarity.

The guide included open-ended questions exploring:

- Biokinetics screening practices for NCD risk factors
- Health promotion and community outreach activities
- Referral pathways and follow-up practices
- Experiences of interprofessional collaboration
- Perceived structural and policy barriers to integration

Following expert review, the guide was pilot tested with one Biokineticist. Subsequently, minor revisions were implemented to enhance the clarity, sequencing, and overall flow of the questions prior to the initiation of data collection. It is important to note that data from the pilot test were excluded from the final analysis. Interviews lasted approximately 45–75 min, were audio-recorded with participants written informed consent, and transcribed verbatim. Field notes were recorded after each interview to capture contextual observations and reflexive insights. All interviews were conducted in English.

### Data analysis

Data were analysed thematically using Braun and Clarke‘s six-phase reflexive thematic analysis framework [26]. This approach was selected for its flexibility and suitability for examining patterned meaning across qualitative datasets.

The analytic process followed six phases:

1. Familiarisation with the data through repeated reading of transcripts
2. Generation of initial codes using both deductive (informed by interview domains) and inductive (data-driven) approaches
3. Collation of codes into candidate themes
4. Review and refinement of themes in relation to the coded extracts and full dataset
5. Definition and naming of final themes
6. Production of the analytic narrative

Transcripts were organised and coded using ATLAS.ti 25. Initial coding was conducted by the first author. Themes were iteratively reviewed and refined through collaborative discussions with co-authors to enhance their analytic depth and credibility. Reflexive memos were maintained throughout the analytic process to document interpretive decisions and emerging conceptual insights. All thematic claims were grounded in verbatim participant excerpts to ensure analytic transparency.

### Trustworthiness and reflexivity

The use of Braun and Clarke‘s six-phase thematic analysis ensured a systematic and transparent analytic process that combined inductive and deductive coding [26]. Throughout the analysis, reflexive practices were maintained to critically examine how the researchers’ professional proximity to Biokinetics may have shaped their interpretation. Given that members of the research team are advocates for the profession, particular attention was paid to ensuring that their advocacy did not overshadow participants’ candid accounts of professional misrecognition, structural exclusion, and interprofessional tension. A clear audit trail was maintained, and all analytic claims were anchored in participant-coded excerpts (P1–P10) to preserve transparency and grounding in the data.

## Results

### Participant characteristics

The study involved 10 participants practising in various provinces within South Africa, each with a median age of 34 and 10 years of professional experience. Most (*n* = 6) participants worked within the South African Military Health Service (SAMHS), which forms part of South Africa’s public sector through the Department of Defence, whereas *n* = 4 participants worked in private practice. Practice locations were mainly urban (*n* = 5) and peri-urban (*n* = 4), with only one in a rural area.

The majority served middle-income clients and frequently managed NCDs such as hypertension, diabetes, and obesity. Regarding resources, half (*n* = 5) reported sufficient access, while three had limited access to essential equipment or facilities. Interprofessional collaboration was common (*n* = 8). Although all participants had experience in rural outreach and/or public-sector contexts, half reported no involvement in community-based health programmes or policy initiatives (see Table 1).

**Table 1.** Participants’ characteristics (*N* = 10).

Characteristic	Summary
Gender	Female (*n* = 6); Male (*n* = 4)
Age (years), median (IQR)	34 (33–40)
Highest qualification	BSc (*n* = 3); BSc (Hons) (*n* = 3); Master’s (*n* = 3); PhD (*n* = 1)
Years of professional experience, median (IQR)	10 (10–14)
Practice sector	Public (*n* = 6); Private (*n* = 4)
Primary practice location	Urban (*n* = 5); Peri-urban (*n* = 4); Rural (*n* = 1)
Access to resources	Adequate (*n* = 5); Moderate (*n* = 2); Limited (*n* = 3)
Interprofessional collaboration	Yes (*n* = 8); No (*n* = 2)

Abbreviations: BSc, Bachelor of Science; BSc (Hons), Bachelor of Science with Honours; IQR, interquartile range; PhD, Doctor of Philosophy.

### Thematic analysis

The thematic analysis of the 10 interviews generated four overarching domains: (1) professional identity and recognition; (2) screening and early detection practices; (3) community-based prevention and management; and (4) structural barriers and opportunities for integration. Together, these domains capture participants’ contributions to NCD detection and prevention, as well as the structural and professional conditions that influence their ability to enact this role in rural healthcare settings. A summary of the domains and key themes is presented in Table 2. Illustrative quotations supporting these themes are presented in Supplementary Table 1.

**Table 2.** Summary of domains and key themes.

Domain	Key themes
Professional identity and recognition	Limited public awareness of Biokinetics; Role confusion with Physiotherapy; Perceived professional tension
Screening and early detection practices	Routine physiological screening; Visual risk communication strategies; Patient behavioural responses to screening; Follow-up and referral challenges
Community-based prevention and management	Outreach and health promotion activities; Cultural and contextual influences; Individualised exercise prescription and supervision; Barriers to sustained engagement
Structural barriers and opportunities for integration	Private-sector dominance and affordability; Limited public-sector posts; Structured military model; Proposed pathways for integration

## Domain 1: professional identity and recognition

### Theme 1: limited public awareness of biokinetics

Participants consistently described limited awareness of Biokinetics in rural communities and reported similar misconceptions among some healthcare professionals, particularly those with limited exposure to the profession. Many described the need to repeatedly explain their professional role.

When I tell people I am a Biokineticist, they don’t understand what that means. Most of the time, they think I am a physiotherapist or that I train athletes. (Female, P3)

Looking at the rural areas, I don’t think people know what Biokinetics is. (Male, P9)

Some participants indicated that this lack of awareness extended beyond their communities to healthcare facilities.

The clinics (in rural areas) don’t even know that Biokineticists exist. (Female, P8)

### Theme 2: role of confusion in physiotherapy

A recurrent theme was confusion between Biokineticists and Physiotherapists among other healthcare professionals. Participants described unclear professional boundaries affecting referrals.

There’s still confusion between Biokinetics and Physiotherapy. (Female, P1)

Other professionals don’t know us … I feel like we are not well recognised. (Female, P5)

Several participants noted that referrals often depended on whether individual doctors understood their scope of practice.

Doctors don’t refer to us because they don’t really know what we do. (Female, P8)

### Theme 3: perceived professional tension

Some participants shared experiences of being viewed as competitors in multidisciplinary environments.

We are seen as a threat … because if patients don’t need them as often, it affects their salary or the pocket. (Female, P4)

This perception was described in the context of referral patterns and interprofessional collaboration.

## Domain 2: screening and early detection practices

### Theme 1: routine physiological screening

All participants reported routinely conducting physiological screening for NCD risk factors. Common measures included blood pressure, glucose levels, cholesterol, body mass index (BMI), and waist circumference.

We always start with vitals: blood pressure, glucose, and waist circumference. These basic checks often reveal risks people didn’t know they had. (Female, P4)

We do questionnaires … medical history, cholesterol, blood pressure, sugar levels, anthropometric measurements. (Male, P2)

Screening typically formed part of an initial assessment prior to exercise prescription.

### Theme 2: visual risk communication strategies

Several participants described using innovative patient-centred communication strategies. These included an informal visual traffic-light coding system to indicate the level of NCD risk. A ‘red’ result for high blood pressure or obesity immediately and visually motivates patients to take action to move towards a ‘green’ status. Furthermore, they explained that these colour-coded systems were developed within their own clinical practice as a simple strategy for communicating cardiovascular risk, rather than being part of formal Biokinetics training.

We profile measurements into green, yellow or red. (Male, P2)

Red means danger … it motivates them to move to green. (Female, P7)

These systems were described as particularly helpful when working with individuals with varying levels of health literacy.

### Theme 3: patient behavioural responses to NCD risk screening

Participants reported varied patient reactions to the NCD risk screening results. Some described patient shock or disbelief upon discovering abnormal values.

They seem to be shocked … like they didn’t expect it. (Female, P8)

Others noted instances of inaccurate or dishonest reporting during lifestyle questioning.

They’ll cheat the test … especially on lifestyle questions. (Female, P8)

In occupational settings, especially industrial work environments, participants described deliberate attempts by patients to manipulate their results.

They’ll take beta blockers or supplements so they can cheat the test. (Female, P8)

### Theme 4: follow-up and referral challenges

While screening by Biokineticists identified individuals at risk, participants reported that these services were a one-off event, especially in community outreach initiatives with inconsistent follow-up.

We give them a report, but there’s no guarantee they’ll go to the clinic. (Female, P5)

Rural outreach contexts were described as particularly affected by limited continuity of care.

In the rural communities we visit, people often can’t afford the taxi fare to the nearest clinic. Even if they know something is wrong, they just live with it. (Male, P2)

## Domain 3: community-based prevention and management

### Theme 1: outreach and health promotion activities

Participants reported conducting health promotion initiatives, including NCD awareness activities through schools, churches, and community presentations, and described embedding exercise education within existing community structures.

We go into churches or schools to do talks and simple aerobics sessions. People respond well; it makes exercise seem less intimidating. (Female, P1)

However, a disparity in clinical rigour emerged, while these community-based sessions were often ‘simple’, workplace screenings and in-house screenings were described as more thoughtfully organised and ‘proper’.

We go to companies and do proper house screening. (Female, P4)

### Theme 2: cultural and contextual influences

Participants highlighted a significant disconnect between cultural norms and the implementation of exercise protocols. Specifically, P5 noted that traditional attire in rural areas acts as a barrier to physical activity, stating

Women aren’t comfortable exercising in their dresses. (Female, P5)

Despite recognising these socio-cultural constraints, the same participant indicated a lack of cultural or religious adaptation in their professional approach, remarking

We do exercise programmes for clients … we don’t include the cultural and religious part. (Female, P5)

### Theme 3: individualised exercise prescription and supervision

Participants underscored the critical role of tailored and progressive exercise regimens in their clinical settings. Specifically, participant 6 emphasised the importance of implementing a phased approach, noting that exercise programs should be initiated gradually and then advance in complexity and intensity in tandem with the patients’ physiological and functional improvements.

We design programmes that start slowly and progress as the patient improves. (Male, P6)

Central to this process is the role of exercise supervision, which participants described as vital for both safety and behavioural adherence. As noted by participant 6, professional oversight facilitates immediate intervention and mitigates the risk of patients performing the exercises incorrectly or prematurely discontinuing.

With supervision, we adjust and keep them on track; without it, they give up or do the wrong things (exercises). (Male, P6)

### Theme 4: barriers to sustained engagement

Participants identified significant structural barriers to sustained engagement, noting a sharp disconnect between patients’ willingness to participate and their practical ability to do so. Financial constraints and geographical distance were cited as the primary drivers of this attrition. As Participant 10 explained, discontinuation is rarely due to poor motivation but rather to a lack of affordable transport and service proximity.

Patients stop not because they don’t want to continue, but because they can’t afford it or it’s too far. (Male, P10)

Participants identified logistical challenges as key factors contributing to the diminished effectiveness of their outreach initiatives. Participant 9 noted a significant gap between expressed interest and actual attendance at community events, stating

You rock up on the day, and 20 people said they were interested, but only two came. (Male, P9)

This disparity underscores the systemic barriers, particularly related to cost and distance, as highlighted by P10, which may inhibit participation despite initial enthusiasm.

## Domain 4: structural barriers and opportunities for integration

### Theme 1: private-sector dominance and affordability

Participants consistently identified the private-sector dominance of Biokinetics as a primary barrier to equity. The profession was described as being financially tied to medical insurance, with Participant 5 noting

Our services can only be paid by medical aid. (Female, P5)

This commercial orientation was further illustrated by Participant 7, who remarked that in the private sector, Biokinetics practice is mainly viewed as a business.

In the private sector, it’s business, not a public service. (Female, P7)

Consequently, participants reported that this model effectively excludes rural and low-income populations who lack the financial means to access private care.

### Theme 2: limited public-sector posts

Participants highlighted that although Biokineticists are employed within SAMHS, a government-funded component of the public sector, comparable employment opportunities remain largely absent from the civilian public health system. Consequently, the benefits of Biokinetics are largely inaccessible to the broader population relying on public PHC, particularly in rural communities.

Outside the army, there’s no public sector space for us. That’s why rural communities don’t see us; the system doesn’t allow it. (Female, P4)

While some participants were aware of small-scale efforts to expand the profession’s footprint, such as the proposed creation of eight hospital-based posts, the overall sentiment was that the public sector remains an untapped and underutilised space for the profession.

They were saying they were creating eight posts in public hospitals. (Male, P6)

### Theme 3: structured military model

Participants working in military healthcare settings reported a well-organised, continuous model of care, which stands in stark contrast to the challenges faced in rural outreach. One participant noted

In the army, we have structured programs with regular follow-up. (Female, P4)

This structured approach effectively addresses the discontinuity that is often seen in other healthcare sectors. Additionally, issues of patient non-compliance were notably absent in this context. Participant 10 commented on the lack of challenges regarding NCD risk screening procedures, stating

For screening, I can’t say that there are challenges. (Male, P10)

These accounts suggest that the controlled and well-resourced nature of the military setting supports a more consistent delivery of Biokinetic services, in contrast to the challenges reported in community-based initiatives.

### Theme 4: proposed pathways for integration

Participants proposed several pathways for integrating Biokinetics into the broader healthcare system, with a particular focus on the public sector. A primary recommendation was the formal appointment of Biokineticists at the PHC level to improve accessibility for underserved populations. As Participant 2 noted

If we had posts in clinics, even once a week, it would make a huge difference in picking up [NCD] risks early and guiding people. (Male, P2)

Furthermore, participants advocated for the inclusion of the Biokinetics profession within the NHI framework. This was presented as a pathway to sustainable economic benefits, with participants arguing that the prevention of NCDs is far more cost-effective than the clinical treatment. Participant 9 emphasised this, stating

NHI should cover Biokinetics because prevention saves costs in the long run. It will be cheaper to invest in us than to treat complications later. (Male, P9)

Collectively, these perspectives position Biokinetics as a valuable component in public health strategy aimed at mitigating the national disease burden. There was a clear consensus on the necessity of embedding Biokineticists within PHC settings and fostering collaborative partnerships with community health workers to enhance the reach and efficacy of preventative care services.

**Figure 1.**
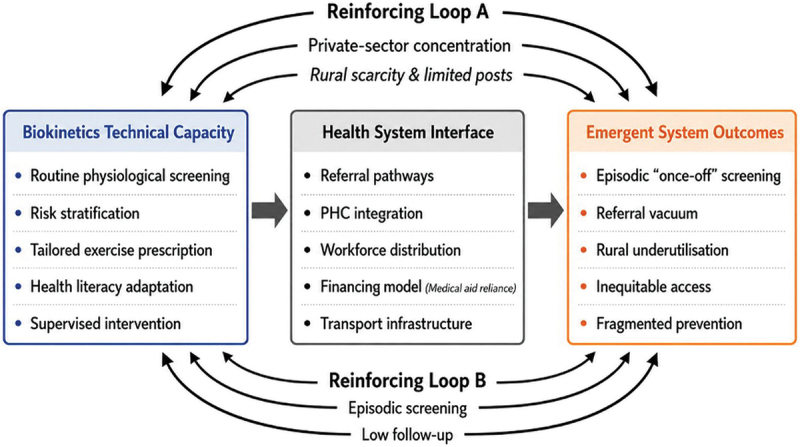
Proposed structural-functional paradox model illustrating how Biokineticists’ technical capacity interacts with health-system constraints to produce fragmented prevention and inequitable access to NCD services in rural South Africa.

To conceptualise these findings, we propose a Structural-Functional Paradox Model (Figure 1). The model illustrates how Biokineticists’ technical capacity for NCD screening and prevention interacts with systemic constraints at the health system interface, producing fragmented and inequitable service deliveries, thereby exacerbating health inequities. Rather than a deficit in professional competence, our findings suggest that NCD prevention efforts are shaped by structural dynamics that limit institutional absorption and rural integration.

## Discussion

This study explored Biokineticists’ perspectives on their current and potential roles in rural NCD detection and prevention, and their experiences of system-level constraints affecting service delivery. Participants consistently described conducting physiological screening for NCD risk factors and delivering tailored exercise interventions, suggesting that they view themselves as clinically capable contributors to early detection and prevention. However, the findings also show a clear structural mismatch between this professional capacity and the broader health system, which limits accessibility, continuity, and equity of care. In rural settings, screening often identifies risk without ensuring sustained management, leaving prevention fragmented rather than continuous [7,18,27].

Participants routinely reported screening for blood pressure, glucose, cholesterol, BMI, and waist circumference, which aligns with national and global recommendations for early detection of cardiometabolic risk in PHC [18,28,29]. Their use of visual communication tools, such as traffic-light coding, also shows professional responsiveness to differing levels of health literacy and supports the role of allied health professionals in communicating modifiable risk and encouraging behaviour change [10,14,30]. These findings indicate that Biokineticists are not simply exercise instructors but clinically oriented allied health professionals with a clear role in early risk identification and behavioural modification [15,17].

At the same time, screening was often episodic. Participants described a ‘referral vacuum’ in which high-risk individuals were identified but then had to navigate overstretched and geographically distant public health services on their own. This is particularly problematic in rural South Africa, where distance, transport costs, and workforce shortages already limit access to cardiovascular and chronic disease services [5,31]. Similar gaps in referral architecture and rehabilitation integration have been reported in South Africa [27] and across sub-Saharan African PHC systems, where NCD integration remains incomplete [7]. In this context, early detection becomes informational rather than interventional, because risk is identified without a reliable pathway to follow-up care [7,32].

A further finding is the private–public access paradox. Biokineticists largely operate within a private-sector model linked to medical insurance reimbursement, while rural populations depend on under-resourced public services [15,17]. This creates a mismatch between where the profession is concentrated and where the burden of NCDs is greatest [24,31]. Participants employed within the SAMHS described structured referral pathways, multidisciplinary collaboration, and continuity of care, suggesting that Biokineticists can be integrated effectively when public systems provide clear pathways and institutional support. Expanding similar models into civilian PHC represents a feasible policy pathway to strengthen NCD prevention, early detection, and multidisciplinary chronic disease management in South Africa [16].

The findings also highlight a disconnection between standardised exercise approaches used by Biokineticists and local cultural practices in rural communities. Participants acknowledged that barriers such as traditional attire limited women’s participation in exercise sessions and recognised that cultural and religious considerations were not routinely incorporated into exercise interventions. These findings suggest that current exercise-based prevention strategies may not always be sufficiently adapted to the cultural, socioeconomic, and environmental realities of rural communities, potentially limiting participation and long-term engagement. Similar observations have been reported in community-based lifestyle interventions in Bangladesh, where culturally adapted physical activity and health promotion programmes achieved greater community participation, acceptability, and sustained behavioural change by aligning interventions with local social and cultural contexts [33,34].

Evidence from culturally tailored physical activity interventions in South Africa demonstrates improved engagement when programmes incorporate local norms, attire considerations, and community structures [35]. Broader African scholarship similarly emphasises that intervention effectiveness depends on contextual adaptation rather than the transfer of standardised protocols [34]. Community-based NCD initiatives that integrate culturally responsive strategies have demonstrated greater sustainability and participation [29]. Therefore, strengthening the contribution of Biokinetics to rural NCD prevention will require not only greater integration into PHC but also the development of culturally responsive and contextually appropriate exercise interventions that align with the lived realities of the communities they are intended to serve.

Collectively, these findings position Biokineticists as an underutilised workforce within rural NCD prevention. Yet the data do not simply argue for ‘more posts’; Rather, they reveal that effective integration requires systemic redesign consistent with health systems strengthening frameworks [7,18,27]:

- Formalised referral algorithms between PHC and Biokinetics
- Public-sector employment pathways
- Multidisciplinary chronic care teams
- Community health worker linkage to mitigate transport barriers [32,36]
- Sustainable financing mechanisms under NHI [18,20,37]

Task-sharing and multidisciplinary PHC models have been identified as key strategies for improving NCD prevention and continuity of care in LMICs [32,36]. Without these structural reforms, screening and outreach efforts will likely remain isolated activities with limited long-term impact. This study contributes to the broader debates on the integration of allied health in LMICs. This demonstrates that workforce capacity alone is insufficient to improve NCD outcomes. Institutional positioning, financing models, and referral architecture determine whether prevention becomes continuous care or episodic detection [7,27]. In this way, the findings extend beyond the profession of Biokinetics. They highlight how health systems that privilege curative, physician-led models may inadvertently marginalise prevention-focused disciplines, even when those disciplines possess the relevant technical expertise [8,18]. Ultimately, strengthening rural NCD prevention will depend not only on expanding the health workforce but also on structurally embedding prevention-oriented allied health professionals within multidisciplinary PHC systems.

### Recommendations

Based on these findings, policymakers should consider integrating Biokineticists into multidisciplinary PHC teams through publicly funded posts and inclusion in NHI service packages. Health workforce planners should develop strategies to improve the distribution of Biokineticists in underserved rural areas, including rural recruitment incentives and, where appropriate, community service placements. Universities should strengthen rural health competencies and expand community-based training opportunities. Future research should evaluate implementation models, including collaboration between community health workers and Biokineticists, and assess the effectiveness and cost-effectiveness of these approaches in improving NCD prevention and continuity of care.

### Strengths and limitations

This study‘s strength is the purposive recruitment of experienced participants in rural outreach and public-sector Biokinetics, aligning with the qualitative aims of in-depth understanding rather than statistical representation. Their extensive experience enabled a detailed exploration of barriers to integrating Biokinetics into rural PHC and early NCD detection. Including diverse participants from various regions and sectors enriched the findings’ credibility. Methods like reflexive thematic analysis and maintaining an audit trail further enhanced trustworthiness.

Several limitations must be acknowledged. The participants, selected for their rural outreach experience, were primarily from urban or military healthcare settings, with only one in a rural area. Thus, the findings primarily reflect the views of experienced Biokineticists, not those of rural PHC professionals or early-career professionals. This purposive sampling feature should be considered for transferability.

Additionally, the study focused solely on Biokineticists’ perspectives, excluding patients, community members, and other healthcare professionals. Consequently, it does not capture the perceptions or experiences of rural communities. Future research should involve multiple stakeholders to achieve a more comprehensive understanding.

Participant self-reports may introduce social desirability bias; however, they openly discussed challenges, indicating their honesty. Credibility was strengthened through open-ended interviews and reflexive techniques.

While the study identified several organisational and health-system barriers, these reflect Biokineticists’ views rather than those of health-system actors or policymakers. Broader stakeholder engagement might be necessary for further exploration.

As with most qualitative research, the findings are context-specific, offering detailed insights rather than broad generalisations. The context descriptions enable readers to evaluate their applicability to other rural, resource-limited healthcare settings facing similar challenges.

## Conclusion

Biokineticists are an underutilised allied health workforce with the potential to enhance early detection and management of NCDs in rural South Africa. However, their contribution is constrained by fragmented referral pathways, low awareness among the public and other professionals, limited public-sector representation, and financing models that favour curative rather than preventive care. Findings from SAMHS participants suggest that Biokineticists can be integrated into multidisciplinary PHC when structured referral pathways and collaborative practice are in place. Addressing these structural and policy barriers could strengthen preventive care and improve access to NCD services in rural settings.

## Supplementary Material

COREQ Checklist.docx

Supplementary materials.docx

## Data Availability

Data may be made available from the corresponding author upon reasonable request and subject to ethical approval.
