# Supplementary material for: Fragmented prevention in rural South Africa: a qualitative study of Biokineticists’ perspectives on health system barriers to early detection of non-communicable diseases
Source: Glob Health Action. 2026 Jul 31;19(1):2707659. doi: 10.1080/16549716.2026.2707659 (PMC13431010; doi:10.1080/16549716.2026.2707659)
Supplement: COREQ Checklist.docx [file ZGHA_A_2707659_SM3145.docx]

| COREQ Checklist (32 items) | | |
| --- | --- | --- |
| Study title: Fragmented Prevention in Rural South Africa: Biokineticists and Health System Barriers to Early Detection of Non-Communicable Diseases | | |
| Domain 1: Research team and reflexivity | | |
| Item | Guide questions | Response |
| Personal characteristics | | |
| 1. Interviewer/facilitator | Which author conducted the interviews? | Interviews were conducted by Gudani G. Mukoma (GGM). |
| 2. Credentials | What were the researcher’s credentials? | GGM holds a BSc (Biokinetics), MSc (Med) and PhD in Paediatrics. |
| 3. Occupation | What was their occupation at the time of the study? | Lecturer in the Department of Biokinetics, Recreation and Sport Science, Faculty of Health Science, University of Venda. |
| 4. Gender | Was the researcher male or female? | Male. |
| 5. Experience and training | What experience or training did the researcher have? | GGM is trained in qualitative research methods and thematic analysis, with experience in conducting in-depth interviews and health systems research. |
| Relationship with participants | | |
| 6. Relationship established | Was a relationship established prior to study? | Some participants were known through professional networks; however, no prior close relationships existed that influenced data collection. |
| 7. Participant knowledge of interviewer | What did participants know about the researcher? | Participants were informed about the researcher’s professional background, study purpose, and interest in NCD prevention and Biokinetics. |
| 8. Interviewer characteristics | What characteristics were reported? | The interviewer is a Biokineticist and researcher, which may have influenced interpretation; reflexive practices were applied to mitigate bias. |
| Domain 2: Study design | | |
| Theoretical framework | | |
| 9. Methodological orientation | What methodological orientation was stated? | Interpretivist paradigm, using reflexive thematic analysis (Braun & Clarke, 2006). |
| Participant selection | | |
| 10. Sampling | How were participants selected? | Purposive sampling with snowball referrals. |
| 11. Method of approach | How were participants approached? | Participants were contacted via professional networks, alumni databases, and referrals. |
| 12. Sample size | How many participants? | Ten Biokineticists (n=10). |
| 13. Non-participation | How many refused or dropped out? | No participants withdrew after consenting; non-response was minimal and not systematically recorded. |
| Setting | | |
| 14. Setting of data collection | Where was data collected? | Interviews were conducted in person or via Microsoft Teams, depending on participant location. |
| 15. Presence of non-participants | Was anyone else present? | No non-participants were present during interviews. |
| 16. Description of sample | What are key characteristics? | Participants were registered Biokineticists with ≥2 years’ experience, working across public, private, and outreach contexts in South Africa. |
| Data collection | | |
| 17. Interview guide | Were questions provided? | Yes, a semi-structured interview guide was developed and pilot tested (Appendix 1). |
| 18. Repeat interviews | Were repeat interviews carried out? | No repeat interviews were conducted. |
| 19. Audio/visual recording | Was recording used? | Yes, interviews were audio-recorded with participant consent. |
| 20. Field notes | Were field notes made? | Yes, field notes were recorded after each interview. |
| 21. Duration | What was the duration? | Interviews lasted approximately 45–75 minutes. |
| 22. Data saturation | Was saturation discussed? | Yes, recruitment continued until thematic saturation was reached. |
| 23. Transcripts returned | Were transcripts returned to participants? | Transcripts were not returned to participants for comment. |
| Domain 3: Analysis and findings | | |
| Data analysis | | |
| 24. Number of data coders | How many coded data? | Initial coding was conducted by GGM, with iterative review by co-authors. |
| 25. Description of coding tree | Was a coding tree described? | Coding followed an iterative process; themes and domains are described in the Results section and Table 2. |
| 26. Derivation of themes | Were themes pre-identified or emergent? | Themes were derived inductively and deductively from the data. |
| 27. Software | What software was used? | ATLAS.ti version 25 was used for data management and coding. |
| 28. Participant checking | Did participants provide feedback on findings? | Participant validation (member checking) was not conducted. |
| Reporting | | |
| 29. Quotations presented | Were participant quotes used? | Yes, illustrative quotations are presented in the Results and Supplementary Table 1. |
| 30. Data and findings consistent | Was there consistency? | Yes, findings are supported by participant quotes and aligned with thematic analysis. |
| 31. Clarity of major themes | Were major themes clear? | Yes, four domains and associated themes are clearly presented. |
| 32. Clarity of minor themes | Were minor themes described? | Yes, sub-themes are described within each domain and supported by data extracts. |
