# Supplementary material for: Fragmented prevention in rural South Africa: a qualitative study of Biokineticists’ perspectives on health system barriers to early detection of non-communicable diseases
Source: Glob Health Action. 2026 Jul 31;19(1):2707659. doi: 10.1080/16549716.2026.2707659 (PMC13431010; doi:10.1080/16549716.2026.2707659)
Supplement: Supplementary materials.docx [file ZGHA_A_2707659_SM3144.docx]

Supplementary materials

Supplementary Table 1. Summary of Domains, Themes, and Illustrative Quotes

| Domain | Theme | Illustrative Quote (Participant) |
| --- | --- | --- |
| Professional Identity and Recognition | Limited Public Awareness | “When I tell people I am a Biokineticist, they don’t understand what that means.” (P3) |
|  | Role Confusion with Physiotherapy | “There’s still confusion between Biokinetics and physiotherapy.” (P1) |
|  | Perceived Professional Tension | “We are seen as a threat… it affects their salary or the pocket.” (P4) |
| Screening and Early Detection Practices | Routine Physiological Screening | “We always start with vitals: blood pressure, glucose, and waist circumference.” (P4) |
|  | Visual Risk Communication Strategies | “Red means danger… it motivates them to move to green.” (P7) |
|  | Patient Behavioural Responses to Screening | “They’ll cheat the test… especially on lifestyle questions.” (P8) |
|  | Follow-Up and Referral Challenges | “We give them a report, but there’s no guarantee they’ll go to the clinic.” (P5) |
| Community-Based Prevention and Management | Outreach and Health Promotion Activities | “We go into churches or schools to do talks and simple aerobics sessions.” (P1) |
|  | Cultural and Contextual Influences | “Women aren’t comfortable exercising in their dresses.” (P5) |
|  | Individualised Exercise Prescription and Supervision | “With supervision, we adjust and keep them on track.” (P6) |
|  | Barriers to Sustained Engagement | “They stop not because they don’t want to… but because they can’t afford it or it’s too far.” (P10) |
| Structural Barriers and Opportunities for Integration | Private-Sector Dominance and Affordability | “Our services can only be paid by medical aid.” (P5) |
|  | Limited Public-Sector Posts | “Outside the army, there’s no public sector space for us.” (P4) |
|  | Structured Military Model | “In the army, we have structured programmes with regular follow-up.” (P4) |
|  | Proposed Pathways for Integration | “If we had posts in clinics, it would make a huge difference.” (P2) |

Supplementary file 1. Semi-structured In-depth Interview (IDI) guide

Introduction

Thank you for agreeing to participate in this study. The purpose of this interview is to explore Biokineticists' perspectives on their current and potential roles in the early detection and prevention of non-communicable diseases (NCDs) in rural South Africa, as well as the health-system factors that influence their integration into primary healthcare. There are no right or wrong answers. We are interested in your experiences and opinions. Everything you share will remain confidential, and you may decline to answer any question or stop the interview at any time.

Section 1. Professional background and perceptions of NCDs

1. Could you briefly describe your professional background and your experience working as a Biokineticist, particularly in rural or public-sector settings?

Probes:

- How long have you been practising?
- What type of practice do you currently work in?
- What experience have you had working with rural populations?

2. From your experience, what are the most important non-communicable diseases affecting rural communities in South Africa?

Probes:

- Which risk factors do you encounter most frequently?
- Why do you think these conditions are common?

3. How do you perceive the role of Biokinetics in preventing and managing non-communicable diseases?

Probes:

- What specific contributions can Biokineticists make?
- Which patient groups benefit most?

Section 2. Screening and early detection practices

4. Could you describe how you usually identify or assess individuals who may be at risk of developing non-communicable diseases?

Probes:

- What screening methods do you use?
- Which physiological measurements are routinely collected?
- How do you assess lifestyle risk factors?

5. How do you communicate screening results and risk information to patients?

Probes:

- Do you use any visual tools?
- How do patients usually respond?
- How do you encourage behaviour change?

6. What challenges do you experience when conducting screening in rural communities?

Probes:

- Resource limitations
- Referral challenges
- Follow-up difficulties
- Patient-related barriers

Section 3. Health promotion and community engagement

7. Could you describe your experience of promoting healthy lifestyles within rural communities?

Probes:

- What health promotion activities have you participated in?
- Have you been involved in community outreach programmes?
- How effective have these activities been?

8. How do you adapt your health promotion or exercise programmes to accommodate the cultural, socioeconomic or environmental realities of rural communities?

Probes:

- Traditional beliefs
- Religious practices
- Financial constraints
- Available facilities

9. What factors facilitate or hinder community participation in your programmes?

Section 4: Management of people living with NCDs

10. Could you describe your role in the ongoing management of individuals living with non-communicable diseases?

Probes:

- Exercise prescription
- Monitoring progress
- Behaviour change support
- Follow-up care

11. How do you collaborate with other healthcare professionals when managing patients with NCDs?

Probes:

- Doctors
- Nurses
- Physiotherapists
- Dietitians
- Community health workers

12. What barriers affect multidisciplinary collaboration?

Section 5: Health system barriers and opportunities

13. From your experience, what health-system factors support or hinder the delivery of Biokinetics services in rural communities?

Probes

- Workforce shortages
- Public-sector employment
- Referral systems
- Funding
- Transport
- Infrastructure

14. How do these factors influence early detection and prevention of NCDs?

15. In your opinion, what changes would most improve access to Biokinetics services within rural primary healthcare?

Probes

- Policy changes
- National Health Insurance
- Public-sector posts
- Community service
- Professional awareness

Section 6. Future directions

16. Looking ahead, how do you see the role of Biokineticists evolving within South Africa's primary healthcare system?

Probes

- Integration into PHC
- Rural healthcare
- Multidisciplinary teams
- Future opportunities

17. Is there anything else you would like to add that we have not discussed but that you believe is important for understanding the role of Biokineticists in preventing and managing non-communicable diseases in rural South Africa?
